# The Importance of Interdisciplinary Care in the Management of Postpartum Hypertensive Crisis

**DOI:** 10.7759/cureus.47423

**Published:** 2023-10-21

**Authors:** Niyati S Upadhyay, Nika Vafadari, Rebecca K Zhang, Joseph Salami, Martin Castaneda

**Affiliations:** 1 Obstetrics and Gynecology, Florida Atlantic University Charles E. Schmidt College of Medicine, Boca Raton, USA; 2 Emergency Medicine, Florida Atlantic University Charles E. Schmidt College of Medicine, Boca Raton, USA; 3 Medicine, Florida Atlantic University Charles E. Schmidt College of Medicine, Boca Raton, USA; 4 Internal Medicine, Florida Atlantic University Charles E. Schmidt College of Medicine, Boca Raton, USA; 5 Obstetrics and Gynecology, Bethesda Hospital East, Boynton Beach, USA

**Keywords:** intensive-care unit, postpartum pulmonary edema, interdisciplinary health team, hypertensive crises, pre-eclampsia, post-partum hemorrhage

## Abstract

Postpartum hypertension can significantly increase maternal morbidity and mortality, and hence it requires prompt interdisciplinary evaluation and interventions. We present a case of a gravid patient with significant comorbidities who required multiple treatments and care from several specialists following a complicated vaginal delivery. The outcome of this case depended on a focused differential diagnosis and interdisciplinary consultation with the several teams involved. This case report illustrates the importance of effective communication and an interdisciplinary approach in the management of postpartum hypertensive emergencies. Such an approach is crucial in reducing maternal complications following postpartum hypertension, as well as reducing the length of hospital stay to improve maternal and fetal outcomes.

## Introduction

Postpartum hypertensive emergencies are defined as an acute elevation of blood pressure ≥160/110 mmHG, any elevation in blood pressure with the development of symptoms consistent with severe preeclampsia, or hypertension associated with end-organ damage such as altered mental status, pulmonary edema, renal failure, or stroke [1]. Prompt intervention is necessary to reduce the chances of the development of eclampsia in the postpartum period, as it is a major cause of maternal deaths in the US. Nearly 10-15% of women with postpartum preeclampsia experience presenting symptoms of eclampsia [2]. It is reported that nearly 70,000 maternal deaths annually are related to preeclampsia- and eclampsia-related complications. In women who develop postpartum preeclampsia, cardiomyopathy leading to chronic heart failure or acute myocardial infarction is one of the most fatal outcomes [2].

Postpartum hemorrhage, maternal obesity, and gestational hypertension are risk factors that may lead to postpartum preeclampsia, which is defined as preeclampsia in the postpartum period within the first 48 hours and up to six weeks after childbirth [3]. Postpartum preeclampsia may be more prevalent in black women, as well as women with high BMI [3]. There is not as strong an association between primigravid women and postpartum preeclampsia as there is with antepartum preeclampsia [3].

Rates of maternal mortality have increased nearly 2.4-fold in the last few decades, reaching a rate of 17.4 per 100,000 live births [4]. While the cause of maternal mortality and morbidity remains complex and a developing field of interest, it is important to be aware of the comorbidities that lead to this higher prevalence. Interestingly, there has been an increase in deaths among women with diabetes mellitus, but a decrease in rates among women with preeclampsia and eclampsia [4]. This decline may be attributed to the growing field of knowledge surrounding peripartum and postpartum complications, and improved prevention and treatment methods for these conditions. Our work aims to supplement these findings by highlighting the importance of interdisciplinary care and effective communication in the immediate postpartum period, especially in the setting of hypertensive emergencies. We hope to further enrich this field of research with a case study that highlights how crucial timely, efficient, and thoughtful inter-team care is in reducing maternal morbidity and mortality rates. We also hope to provide insights into the importance of standardizing care for postpartum hypertensive emergencies through an algorithmic approach.

## Case presentation

A 26-year-old, obese, primigravid black woman with gestational diabetes mellitus A2 and gestational hypertension was admitted to the labor and delivery unit for induction of labor at 39 weeks following a diagnosis of gestational hypertension. Her blood pressure and creatinine on admission were 139/91 mmHg and 0.65 mg/dL, respectively. Her hemoglobin on admission was 11.8 g/dL. She was successfully induced with Cervidil 10 mg and Pitocin 30 units and she maintained category I tracings throughout her labor. Her labor course became complicated at the time of delivery due to shoulder dystocia. The McRoberts maneuver was performed and suprapubic pressure was applied, with successful delivery of the posterior shoulder. She delivered a male infant weighing 8 lb and 4 oz, with Apgar scores of 8 at one minute and 9 at five minutes. The patient was noted to have postpartum hemorrhage secondary to uterine atony following delivery. She received four doses of Hemabate in total as well as tranexamic acid 1000 mg. Then, vigorous uterine massage was performed and normal uterine tone was achieved.

The patient then became hypertensive and hypoxemic after delivery. She was started on supplemental O_2_ via nasal cannula, magnesium for neuroprotection, and intravenous labetalol 20-40 mg for blood pressure control. On physical examination, her breath sounds were diminished bilaterally. Pregnancy-induced hypertension (PIH) labs were drawn and they showed creatinine of 1.2 mg/dL, at which point magnesium was discontinued and Keppra was started. The patient's clinical picture and blood pressure remained stable after this intervention.

**Figure 1:**
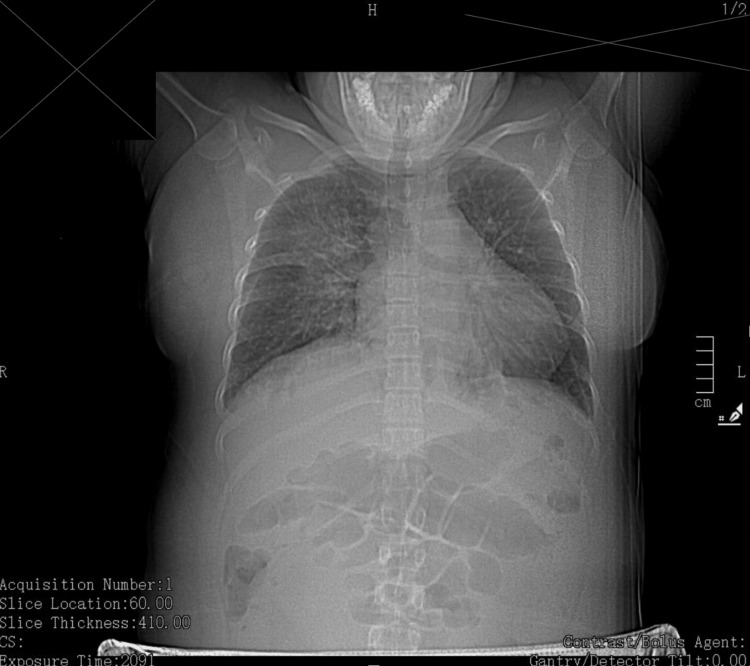
Chest CT scan showing right-sided perihilar infiltrate and bilateral pleural effusions (right > left) CT: computed tomography

On postpartum day one, the patient's clinical picture began to worsen. Her blood pressure was elevated at 140/104 mmHg and she became increasingly short of breath. The creatinine level was 1.04 mg/dL, but there was no evidence of proteinuria. Her hemoglobin dropped to 8.9 g/dL. Intravenous labetalol was initially declined by the patient but was eventually administered later that evening at a dose of 20 mg with appropriate blood pressure control. High-sensitivity troponin and B-type natriuretic peptide (BNP) were elevated at 318 ng/L and 242 pg/mL, respectively. A chest CT (Figure 1) was subsequently performed due to growing concerns of peripartum cardiomyopathy and pulmonary embolism and showed bilateral pleural effusion and extensive right-sided perihilar infiltrate suspicious for pneumonia, for which an infectious diseases specialist was consulted for appropriate pharmacotherapy.

**Figure 2:**
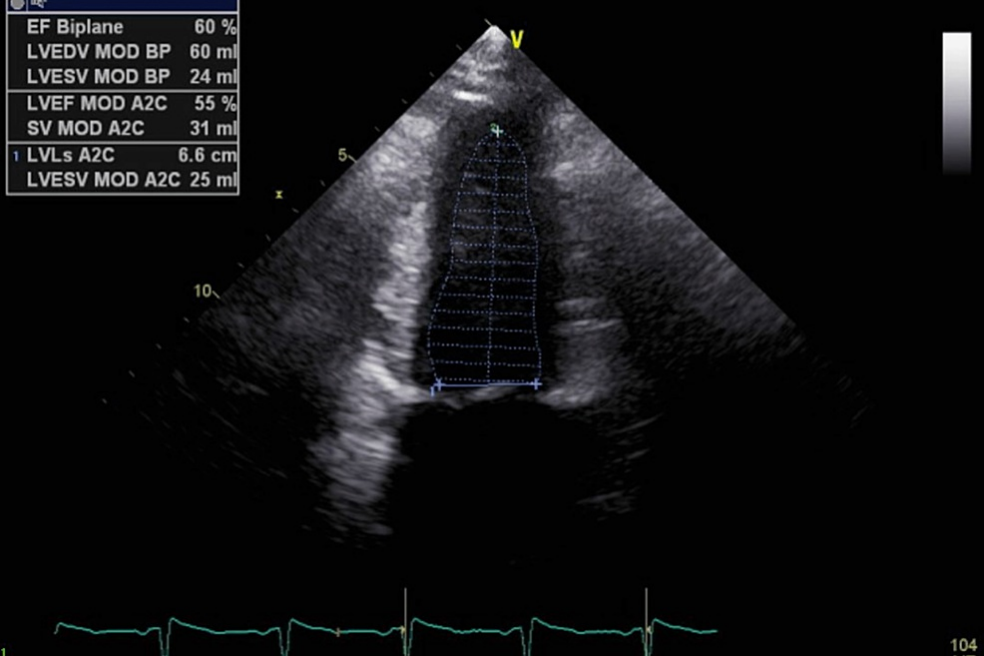
Echocardiogram showing ejection fraction of 55-60% with no evidence of thrombus or atrial or ventricular enlargement

**Figure 3:**
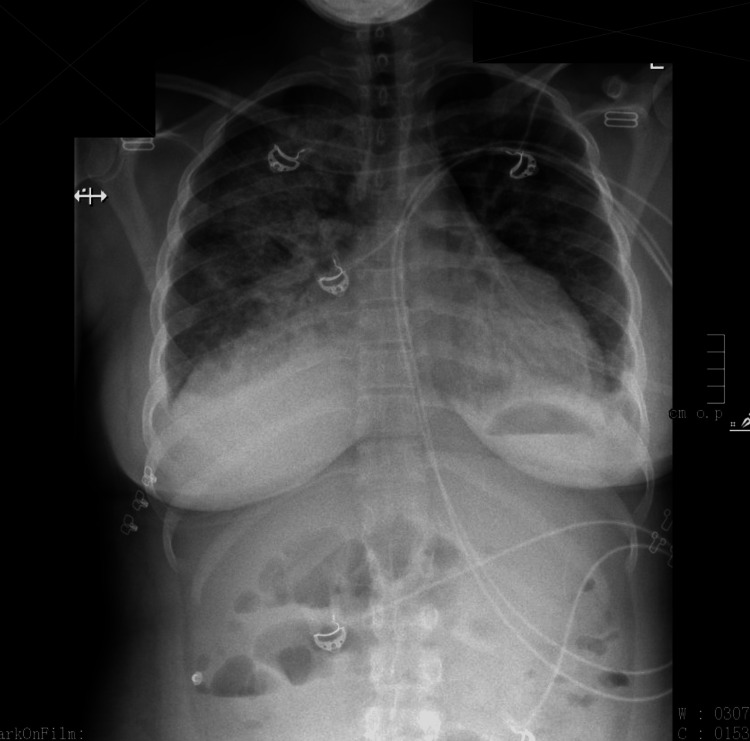
Chest X-ray showing bilateral pleural effusions (right > left) and right-sided perihilar infiltrate with pulmonary opacities The findings are compatible with pulmonary edema vs. lobar pneumonia

The patient's respiratory status worsened and she was subsequently started on bilevel positive airway pressure (BiPAP). The intensivist was consulted, and the patient was admitted to the ICU for further management. BiPAP was continued, as well as intravenous labetalol for BP control, intravenous Lasix 20 mg for diuresis, and broad-spectrum antibiotics (cefepime, azithromycin, vancomycin, and ampicillin). Cardiology was consulted, and an echocardiogram (Figure 2) was performed, which showed normal left ventricular structure and function. A chest X-ray was also done at this time (Figure 3). The patient's presentation was consistent with acute pulmonary edema secondary to preeclampsia with severe features.

The patient’s symptoms and vitals eventually improved on hospital admission day five, and she was weaned off BiPAP, transitioned to oral antihypertensive medications, and transferred to the inpatient floor. Her blood pressure on discharge ranged from 120-137 mmHg systolic over 70-89 mmHg diastolic. She was discharged in stable condition and was sent home with oral antihypertensive medications, with instructions to follow up in four weeks for a postpartum evaluation.

## Discussion

Some of the most common etiologies of postpartum hypertension (beyond 48 hours after delivery) include preeclampsia, eclampsia, and chronic hypertension that existed before pregnancy [5]. Postpartum hypertensive emergencies can lead to serious maternal outcomes such as cerebrovascular accidents, cardiomyopathy and congestive heart failure, renal failure, and even death [5]. Beyond elevated blood pressure, the healthcare team should be aware of symptoms associated with the poor outcomes of postpartum hypertension. These include but are not limited to dyspnea, orthopnea, tachycardia, chest pain, headache, and visual changes [6]. Physical exam findings that may raise concerns include absent/distant breath sounds, crackles, jugular venous distension, and altered mental status. It is crucial to recognize the signs of potentially fatal outcomes in the postpartum period, especially in a patient with a new-onset hypertensive emergency. It is also important to be aware of the risk factors that may predispose a patient to developing postpartum hypertension or preeclampsia. As previously mentioned, black race and maternal obesity [3] are risk factors for postpartum preeclampsia and were both present in this case. Prenatal counseling on the importance of a balanced diet and exercise could play a crucial role in preventing or reducing some of these adverse outcomes.

The current recommendations for the treatment of postpartum hypertensive emergencies typically follow a stepwise algorithm [7,8]. In general, first-line management of blood pressure should be accomplished within 30-60 minutes of onset with intravenous labetalol or hydralazine. Oral nifedipine may be used if intravenous access is limited. Beyond this, management depends on the patient’s history and clinical presentation. In cases like the one presented, the patient’s clinical picture and physical exam findings dictate the need for further imaging and consultations with various specialists. This algorithm in the management of postpartum hypertension [5] emphasizes the need for multidisciplinary care that is guided by clinical presentation. This approach recognizes the need for specialists and consultants but notes that these will vary based on the patient’s history and presenting symptoms. This case report highlights the importance of stepwise evaluation and management of this type of emergency. 

The existing literature on postpartum care to reduce maternal mortality is limited but growing. Walker et al. [6] have recommended the consolidation of guidelines for members of the interprofessional team - nurses, midwives, and physicians - to augment a woman’s recovery in the postpartum period. This work focuses on effective communication and education of all providers to assess warning signs in postpartum complications. They created a guideline for teaching team members about immediate postpartum symptoms to be familiar with, as well as a tiered communication system. Our work aims to contribute to this field of research by showing how crucial effective communication is in the postpartum period. We hope to provide a viewpoint that supports the need for more training and simulations to enable members of the healthcare team to familiarize themselves further with the management of postpartum hypertensive emergencies.

A patient such as the one presented in this case would benefit from pre-conception counseling and peripartum counseling on comorbid conditions that may affect her pregnancy and delivery. While this was not provided in this case, it can play a crucial role in preventing severe outcomes or preparing the delivery team to deal with a potential adverse outcome.

## Conclusions

The adverse effects of postpartum hypertensive emergencies are numerous and can impact several organ systems. Early detection, recognition, and intervention are necessary to reduce fatality in the postpartum period. This case report highlights the need for effective intervention involving multiple medical specialties in cases of postpartum hypertensive emergencies. Our case report documents one of the many successful instances that benefitted from the timely and coordinated intervention in postpartum hypertensive crises. Effective communication strategies and algorithmic approaches to hypertensive emergencies should be implemented across the board to help reduce fatal outcomes in these patients.
